# A principle of organization which facilitates broad Lamarckian-like adaptations by improvisation

**DOI:** 10.1186/s13062-015-0097-y

**Published:** 2015-12-02

**Authors:** Yoav Soen, Maor Knafo, Michael Elgart

**Affiliations:** Department of Biological Chemistry, Weizmann Institute of Science, Rehovot, 76100 Israel

## Abstract

**Background:**

During the lifetime of an organism, every individual encounters many combinations of diverse changes in the somatic genome, epigenome and microbiome. This gives rise to many novel combinations of internal failures which are unique to each individual. How any individual can tolerate this high load of new, individual-specific scenarios of failure is not clear. While stress-induced plasticity and hidden variation have been proposed as potential mechanisms of tolerance, the main conceptual problem remains unaddressed, namely: how largely non-beneficial random variation can be rapidly and safely organized into net benefits to every individual.

**Presentation of the hypothesis:**

We propose an organizational principle which explains how every individual can alleviate a high load of novel stressful scenarios using many random variations in flexible and inherently less harmful traits. Random changes which happen to reduce stress, benefit the organism and decrease the drive for additional changes. This adaptation (termed ‘*Adaptive Improvisation*’) can be further enhanced, propagated, stabilized and memorized when beneficial changes reinforce themselves by auto-regulatory mechanisms. This principle implicates stress not only in driving diverse variations in cells tissues and organs, but also in organizing these variations into adaptive outcomes. Specific (but not exclusive) examples include stress reduction by rapid exchange of mobile genetic elements (or exosomes) in unicellular, and rapid changes in the symbiotic microorganisms of animals. In all cases, adaptive changes can be transmitted across generations, allowing rapid improvement and assimilation in a few generations.

**Testing the hypothesis:**

We provide testable predictions derived from the hypothesis.

**Implications of the hypothesis:**

The hypothesis raises a critical, but thus far overlooked adaptation problem and explains how random variation can self-organize to confer a wide range of individual-specific adaptations beyond the existing outcomes of natural selection. It portrays gene regulation as an inseparable synergy between natural selection and adaptation by improvisation. The latter provides a basis for Lamarckian adaptation that is not limited to a specific mechanism and readily accounts for the remarkable resistance of tumors to treatment.

**Reviewers:**

This article was reviewed by Eugene V. Koonin, Yuri Wolf and Itai Yanai.

## Hypothesis

### Background

#### Requirement and evidence for adaptive improvisation during ontogeny

It is commonly accepted that stable adaption to new environments is mediated mainly by natural selection of individuals carrying advantageous genetic changes. This view distinguishes the long term evolutionary process of genetic adaptation from physiological adaptation that may take place within generation. The latter is often viewed as ‘execution’ of previously evolved programs. This strategy of physiological adaptation is mostly effective for coping with conditions that have been encountered repeatedly during the evolutionary history of the organism (e.g. exposures to heat, starvation, drought, predation, etc.). However, in the case of rare or completely novel scenarios of stress, the organism is unlikely to have had sufficient prior opportunities to evolve specific programs of adaptation. Whether and how organisms can still mount adaptive responses in these cases is largely unknown.

The requirement for an ability to cope with novel scenarios of stress is not limited to cases of evolutionarily novel environments (which may indeed be rare). Even in highly regular environments, every individual is expected to encounter many individual-specific combinations of internal changes which can compromise the otherwise beneficial outcomes of pre-evolved mechanisms. These include, for example, somatic genetic aberrations in every cell, epigenetic changes in cells, microbial changes within the organism, etc. Additionally, changes within a cell of a particular tissue may impact other cells within and outside this tissue, thus leading to co-existing aberrations at multiple scales (from intracellular process to the whole organism and its environment). Since the combinatorial space of these changes is immense, an overwhelming fraction of the specific combinations of changes could not have been sampled during evolution. Of course, many of the non-sampled (i.e. novel) changes can be dealt with by highly efficient, pre-evolved generic mechanisms. For example, genetic adducts are efficiently removed by DNA repair mechanisms [1], aberrant cells are eliminated by apoptosis [2, 3], immune surveillance [4–8] and/or additional mechanisms [9], and pathogenic micro-organisms are contained or eliminated by the immune system [10–13]. However, these generic mechanisms cannot provide complete protection against all scenarios of disrupted homeostasis [14–26]. For example, the immune system successfully eliminates many viruses, but some of them manage to evade this system using a variety of approaches [27–31]. Such viruses have been demonstrated to substantially alter transcription in cells of the infected host [32, 33] and can generate novel functional transcripts [34–37]. Since almost all living organisms contain a multitude of viruses which modify the host and evolve at a greater pace than the host genome [38–41], there can be no genetic program for handling all the possible scenarios of maladaptive conditions. A similar limitation applies to coping with individual-specific epigenetic drifts associated with aging [42–44] and recovering from other types of individual-specific lesions (e.g. microinfarcts in various tissues [45, 46]). This shortcoming is reflected in many cases of dysfunction that have individual-specific components, such as chronic illnesses, auto-immunity and tumorigenic transformations.

It is generally accepted that coping with novel scenarios of severe stress requires phenotypic plasticity [47–56] and may involve exploratory processes [56, 57]. However, without mechanisms that have been previously selected based on their ability to mount beneficial responses to individual-specific conditions, it is not clear how plastic changes and exploratory processes can be adaptive. In fact, in the absence of efficient ways to bias plastic changes towards beneficial outcomes, plasticity is expected to do more harm than good (much like random mutations). This problem cannot be addressed by natural selection, which operates at the (large) population level, but is ineffective when each individual encounters a large number of individual-specific perturbations ('failures') due to internal changes. Put differently, mutations which could protect some individuals under rare external stress do not assist in coping with a multitude of individual-specific combinations of internal failures. Phenotypic robustness is also not effective enough because robust phenotypes may become maladaptive in a non-negligible fraction of the internal failures. A qualitatively different strategy of (‘quasi-Lamarckian’) adaptation to novel environment [58, 59] has recently been inspired by experimental evidence of stress-induced mutagenesis [60–68]. It proposed that environmental conditions trigger non-specific mutations which confer adaptation to the stress-inducing factors [59]. The deleterious potential of such random mutagenesis was thought to be addressed (in prokaryotes) by regulation which involves DNA repair and particular structure of genome architecture. This regulation is presumed to bias mutations in a way that allows coordinated evolvability of functionally linked genes in rare cells where beneficial mutations emerge [59]. While this could indeed benefit rare cells in novel environments, it does not address the conceptual problem that is mentioned above, namely: how any regulation could use mostly non-beneficial variation to provide every individual with some capacity to make beneficial use of this variation in arbitrary types of novel conditions. Addressing this problem becomes far more challenging when each multicellular individual has to cope with many (individual-specific) combinations of internal perturbations in different cells, tissues and organs. Notably, the main question behind this adaptation problem is not how stress induces variation (or plasticity), nor how this variation can be transmitted across generations and whether it can be beneficial to some individuals under rare novel environments. The question is what kind of physiological regulation could facilitate *de novo* emergence of many multi-scale adaptations using random processes that are largely non-beneficial.

The importance of this question cannot be over-estimated. If every individual encounters many new scenarios of internal failures due to combinations of genetic and non-genetic changes, it is plausible that no individual can exist without some capacity to organize random variation in a beneficial manner. Lack of it may therefore prevent a population from even forming, thus invalidating natural selection altogether. This capacity, however, does not (and cannot) replace mechanisms that were established by natural selection, but rather complement these mechanisms.

### Conceptual difficulties which must be addressed

Any conceptual mechanism which makes beneficial use of random changes must address the following critical questions:

- How the induction of random changes could avoid disrupting essential processes which took a very long time to evolve?
- How beneficial random changes could be identified within the immensely large space of non-beneficial changes? Here, we should bear in mind the relatively short time available to find viable solutions (less than one generation time) and the probable necessity of testing many putative solutions.
- How random changes in different cells, tissues and organs can be coordinated into beneficial outcomes for the whole organism? For example, in the case of animals (as opposed to single cell organisms), it is not enough that every stressed cell will ‘find’ its own solution, because this may lead to non-coordinated (tumor-like) changes that severely compromise the organism as a whole.

In addition to these critical questions, it would be desirable (albeit not necessary) to propose a conceptual mechanism which is compatible with transgenerational inheritance of some of the newly-formed beneficial changes. Such inheritance would enable progressive improvement of the adaptation over generations. This should not be confused with examples of Lamarckian adaptation by pre-evolved mechanisms which address a specific type of novelty. Hallmark examples of these Lamarckian mechanisms are the CRISPR system in prokaryotes [69–71] and the small RNA-based mechanism of viral immunity in *C. elegans* [72]. Both mechanisms enable rapid acquisition of heritable resistance to new viruses, but the tolerance is always acquired using the same mechanistic pathway. While the preexistence of such pathway is very instrumental for coping with new viruses, it does not provide solutions for all other types of novel conditions (including pathogenic outcomes of new viruses that have managed to evade rapid elimination).

### Evidence supporting a capacity to adapt by newly forming random changes

Potential signatures of a capacity to use random variation for generating individual-specific adaptations may be recognized in diverse contexts: Adaptive immunity in human incorporates ongoing generation of genetic changes in ways which permit adjustments of responses to co-evolving pathogens during the lifetime of a single person [73]. Similarly, the primate brain exploits neural learning for coping with new pathophysiological and intellectual problems. A striking example for this is given by *de novo* reorganizations of cortical motor neuron activities in ways which enable acquisition of control over a prosthetic arm [74]. Electrical stimulation of neural networks in a dish reveals analogous capacity for *de novo* learning in arbitrary configurations of stimulated neurons [75, 76]. A classic example in a developmental context was provided by a two-legged goat, born with a congenital paralysis preventing the use of the front legs [77]. This condition led to re-organization of anatomical features enabling hopping on the hind limbs [47, 53]. Functional reorganization of developmental processes is also apparent in various cases of mating between different species or breeds, in which distinct ‘programs’ are merged into functional outcome without a specific 'program of merging'. These cases include heterosis [78], plant grafting [79], mating of pure-bred dogs which differ substantially in their skull size and shape ([80], page 556) and even cross-genus cloning of one species into another [81]. The capacity to form new adaptations within one or few generations is not at all limited to multicellular organisms. It was clearly demonstrated by synthetic gene recruitment in yeast which de-coupled an essential gene (*His3*) from its endogenous regulation and placed it under a non-related promoter (GAL4). Repression of the GAL4 promoter by switching to glucose-based medium drove rapid adaptation [82] which varied substantially between replicated experiments [83, 84] and did not necessarily involve genetic changes [85]. An additional mass of supportive (though less explicit) evidence in non-engineered settings of micro-organisms is provided by rapid acquisition of non-coding DNA and mobile genetic elements in bacteria [86–89], which may account for their surprisingly high rate of acquisition of anti-viral and antibiotic resistance [59, 87–89].

### Presentation of the hypothesis

#### Hypothesis

Coping with novel scenarios of stress is enabled by a large number of readily changing (i.e. ‘flexible’) traits or processes (collectively referred to as traits or features), combined with a sufficiently strong inverse-correlation between the flexibility of traits and their deleterious potential. Adaptation is then achieved by occasional stress reduction due to random changes primarily in those flexible traits and processes (‘*Adaptive Improvisation*’) and is further enhanced and stabilized by auto-regulation. Herein, we operationally define adaptation (or beneficial variation) as any change in the organism which reduces stress. Without a sufficiently strong inverse-correlation between flexibility and deleterious potential, the probability of deleterious outcomes would have been too high because most of the random variation is not beneficial.

This organizational concept is based on the following assumptions:

### Underlying assumptions

1. *Wide distribution of flexibilities*: The traits and processes of each individual cell and organism have different capacities to vary in response to external or internal perturbations. Some traits are tightly regulated and are kept within a small margin of change (stable or ‘strongly constrained’ traits). Others are much more ‘flexible’ (weakly constrained) and can vary significantly and rapidly during the lifetime of the individual. These traits can include: a) expression, activity, location and affinity of non-essential genes or regulatory RNA, b) conformations of intrinsically unstructured proteins, c) abundance and exchange of non-essential mobile elements, and d) any other type of change that is not constrained by tight regulation. We assume that the distribution of flexibilities is very wide, so that every individual has a very large space of flexible features. A qualitative example of a putative distribution is illustrated in Fig. 1.

**Fig. 1:**
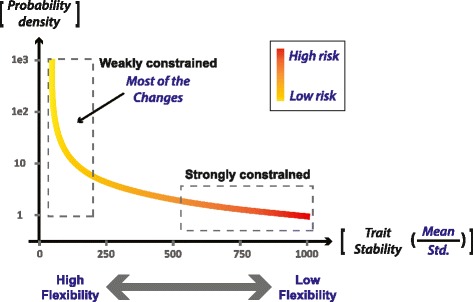
Putative distribution of (trait) flexibility and deleterious potential (risk level). The probability density curve corresponds to the density of traits (y-axis) at a given level of stability (x-axis). The flexibility of a trait (1/Trait Stability) is represented by the standard deviation, divided by the mean (*Std./Mean*), both computed over time in a single individual. The expected density of traits is an increasing function of trait flexibility and a decreasing function of the trait’s deleterious potential (risk level). We also expect a wide range of flexibility values, illustrated in this example by a (scale-free) power-law increase of the density as a function of trait flexibility. The inverse correlation between the flexibility of a trait and its deleterious potential is represented by a color code, with red (‘high risk’) and yellow (‘low risk’) associated, respectively, with high and low probabilities for a detrimental outcome of a change in the respective trait
2. *Inverse connection between flexibility and deleterious potential*: A change in a flexible trait tends to be less detrimental compared to similar magnitude of change in a highly constrained trait (Fig. 1). This assumption is a direct outcome of natural selection, which filters out those individuals in which deleterious changes are not prevented by stringent regulation.
3. *Reciprocal feedback between stress and random changes*: Novel conditions induce stress which drives modifications in various traits. The probability of inducing random variation in a particular trait increases with the degree of flexibility of this trait and the strength of the stress. Consequently, the stress is inducing changes primarily in flexible traits [90]. Combined with assumption 2, these changes cause less harm than a similar magnitude of change in more constrained feature.

### Elements and properties of adaptation by improvisation

Based on the above assumptions, we propose a general process of emergent adaptations by improvisation. This process can involve any type of mechanism, thus providing broad adaptive capacity which is not exclusively dependent on a specific mechanism. The core component of this adaptation is the alleviation of stress by drive reduction. While this might be sufficient for adaptation by improvisation, the outcome of it can be enhanced and stabilized by auto-regulation.

A. **Alleviating stress by drive reduction**

**Fig. 2:**
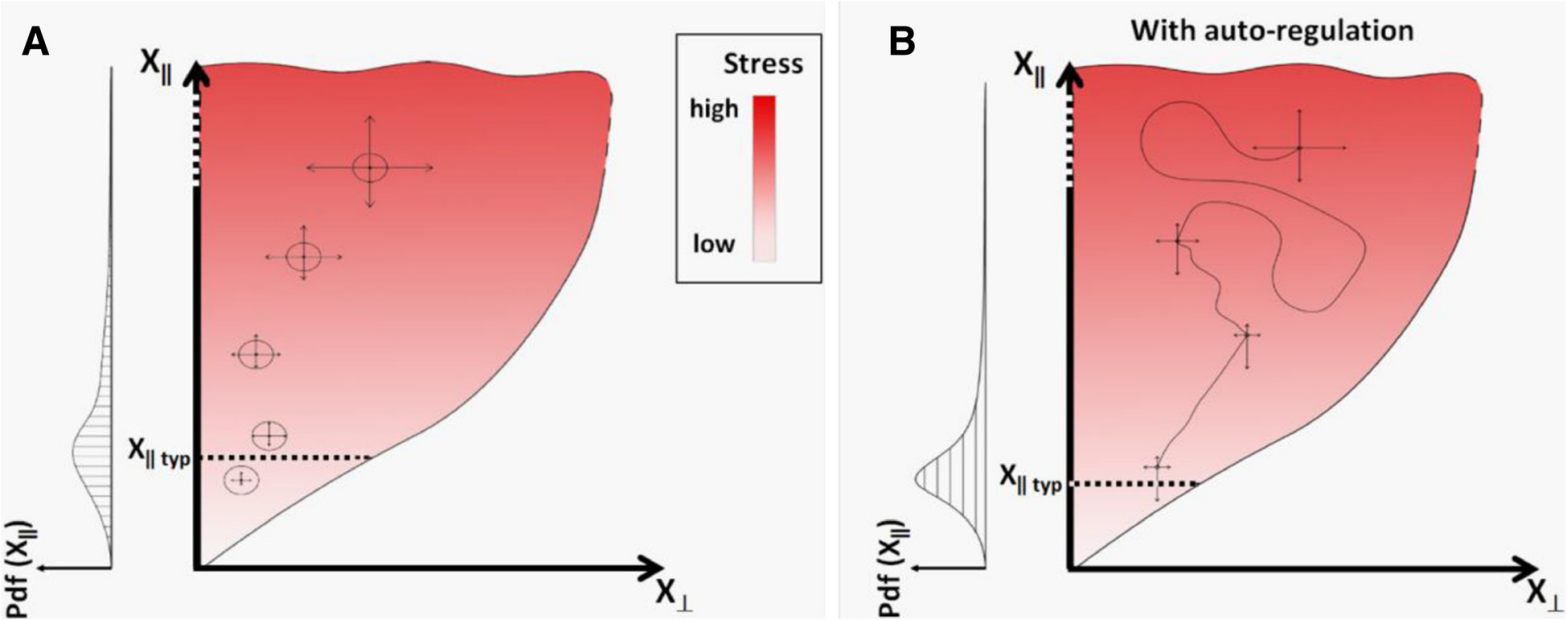
A visual metaphor illustrating adaptive improvisation by random drive reduction. Improvisation (or exploration) is defined as a change in state (or part of the change) which is not specified by pre-evolved mechanisms. Improvisation which reduces the stress is termed 'adaptive' (or ‘beneficial’). Illustrations with and without re-enforcement are shown in (a) and (b), respectively. **a** Heuristic depiction of the state space available to the organism (colored area). At any given moment, the state is represented by a high dimensional vector X, which specifies a point in the available state space. The overall amount of stress, S, at a given state, is displayed in red color code (for simplicity of the illustration, we only consider here a single measure of stress at the whole organism level). The available state space is divided into two subspaces, X_**ǁ**_ and X_**⊥**_, defined as follows: Changes in state upward along X_**ǁ**_ increase the overall stress while changes along X_**⊥**_ have no effect on the stress (‘no effect’ means that the change in stress is below a small threshold). Thus, the stress S in this representation, is an increasing function ƒ of the state along X_**ǁ**_, i.e. S = ƒ(X_**ǁ**_). Additionally, the characteristic magnitude of exploratory changes (|ΔX| _char_) is assumed to be an increasing function *G* of the stress, i.e. |ΔX| _char_ = *G*(S). Consequently, the lower the stress, the smaller the ‘perimeter’ of subsequent exploration, and the state of the organism is more likely to remain (over a specified time interval) within a given neighborhood of the starting state (circle). This biases the outcome towards less stressful states, even without directed movements in the state of possible changes. The tendency toward lower stress is counteracted by having a smaller number of stress-reducing states compared with states associated with increased stress. The balance between the tendencies to decrease and increase the stress depends on the characteristics of *G*(S) and the relative abundance in the number of stress-reducing versus stress increasing states. We assume that in the regime of very high stress, the over-abundance of stress-increasing states becomes small and the overall balance would favor decrease in stress due to drive reduction. However, in the inverse regime of very low stress, states which increase the stress are much more abundant than stress-reducing states and the exploratory changes will tend to increase the stress. The combined effects of upward and downward tendencies create an intermediate domain, in which the organism is most likely to be found (around X_ǁ typ_). A qualitative profile of a probability density function (Pdf) for a particular state along X_**ǁ**_, is shown to the left. **b** Amplification and stabilization of beneficial changes by auto-regulatory processes. Random occurrence of beneficial processes that are also capable of re-enforcing themselves (or each other), enhance the process of stress reduction thus increasing the benefit. Since the resources of every system are limited, activation of beneficial auto-regulatory processes tend to repress other processes, thereby stabilizing the beneficial processes. Subsequent changes, in this case, become more likely to decrease stress (indicated by substantially asymmetric arrows), thus expediting the adaptation, stabilizing its outcomes and reducing the probability of more harmful changes

Any stress which is not sufficiently alleviated by existing (pre-evolved) stress responses drives rapid (not necessarily genetic) changes mostly in flexible and relatively non-harmful features. These changes can potentially alleviate the stress, but can also aggravate it. However, changes in directions which alleviate the stress (operationally defined as adaptive/beneficial), also reduce the drive for additional changes, thus promoting higher persistence of states (and processes) associated with lower stress (Fig. 2a). On the other hand, states which aggravate the stress increase the magnitude of additional changes, which then tend to drive the organism more strongly away from these states (Fig. 2a). This creates a statistical bias towards establishment of stress-reducing (i.e. beneficial) changes. This bias can be viewed as an extension of Le Chatelier’s principle [91], from chemical systems to biological self-organization. In this framework, stress provides not only a drive for variation but also a means for relaxation.

Since stress can be induced at (and have an impact on) every organizational level (e.g. cellular, tissue/organ and the whole organism level), stress-reductions can occur in multiple levels, enabling *de novo* emergence of coordination between adaptive improvisations in different cells, tissues and organs. As a basic principle of organization, it also applies to higher levels of organization (e.g. populations of individuals), but our main concern here is the organization within an individual.

Notably, the level of stress cannot be reduced to zero, because the over-abundance of changes which increase stress becomes stronger with decreased stress (Fig. 2a). At sufficiently low levels of stress, this over-abundance outweighs the effect of drive reduction, and random changes then lead to increase of stress. The most expected levels of stress therefore reside in an intermediate regime (indicated by the distribution function in Fig. 2a) in which the drive-reduction effect is roughly balanced by the entropic effect of higher abundance of stress-increasing states.

B. **Enhancement and stabilization of stress-reduction by auto-regulation**

Stress alleviation by drive-reduction is further enhanced and stabilized when beneficial changes are also capable of reinforcing themselves by positive feedback mechanisms (Fig. 2b). An obvious example is provided by auto-regulatory processes in which beneficial changes are conferred by factors that are also capable of promoting their own production. Due to limited resources available to the organism, enhancement of these auto-regulatory changes can repress other (not necessarily beneficial) alternatives, thus leading to further selection and stabilization of the beneficial changes. This can expedite the process of coping with the stress, stabilize beneficial outcomes, and reduce the probability of more harmful changes. Stabilization by auto-regulation may also contribute to the persistence of the adaptive changes even when the inducing conditions revert, thus providing potential for maintaining these changes as ‘memorized’ adaptations that are immediately available upon re-introduction of the stressful conditions. Moreover, inter-individual exchange of beneficial auto-regulatory factors can reduce the level of exploration performed by each individual, thereby decreasing the potential for harmful changes in these individuals.

C. **Additional considerations**

*Natural ‘division-of-workload’ between defined and exploratory stress responses*

Stress can be reduced either by pre-evolved response programs or via the much slower process of adaptive improvisation. The relative contribution of each process is determined by the efficacy of the response, without any need for a computational-like ‘decision making’ processes. If the stress is ‘familiar‘ (e.g. heat shock, starvation, drought, etc.) and sufficiently moderate, it is quickly recognized by existing mechanisms and invokes efficient pre-evolved responses which rapidly alleviate the stress. This lowers the drive for random changes, thereby reducing the extent of adaptive improvisation. However, when the stress is novel or too strong, its alleviation by pre-evolved responses is compromised and the remaining stress increases the extent of adaptive improvisation.

*Balance between beneficial and deleterious outcomes*

A purely random variation has two opposing influences: It increases the probability of finding a solution to a new problem (adaptation), but also the likelihood of encountering deleterious changes. The adaptive potential of random variation is therefore expected to depend on the ratio between the probabilities of beneficial and deleterious outcomes of a given change:

$$ Adaptive\  Potential \sim \left[\  Prob.\ \left( beneficial\  outcome\right)\ \right]\ /\ \left[\  Prob.\ \left( deleterious\  outcome\right)\ \right] $$

To enable beneficial use of random variation, this ratio must be substantially higher than one. This requires a strong inverse correlation between the flexibility of traits and their deleterious potential (Fig. 1). The inverse correlation is an intrinsic outcome of natural selection which lowers the flexibility in directions that have been previously deemed deleterious. Due to this bias, random changes occur primarily in less deleterious traits, thus reducing the detrimental effect of the changes. The probability for beneficial (adaptive) random changes, on the other hand, is not preferentially dependent on the ability to vary along the more restricted directions. Consequently, the restriction of variation in critical traits suppresses deleterious changes much more than it prevents beneficial changes. The stronger the differential suppression of deleterious potential, the larger the bias towards less harmful random changes. A sufficiently strong bias reduces the detrimental effects enough to enable stress reduction (i.e. adaptation) by newly-forming random variation. However, it is important to realize that this bias towards beneficial random variation cannot guarantee adaptive outcome. No matter how large the bias is, it does not necessarily lead to adaptation by improvisation because the probability of non-beneficial changes cannot be reduced to zero. In other words, adaptive improvisation is a likely outcome, but not a guaranteed outcome.

The ability to restrict random variation has been suggested to occur even in genomic space [58]. The usefulness of this restriction depends, however, on the ability to differentially suppress changes in loci with high deleterious potential. Achieving this for genetic changes requires functional separation of the genome into readily changing elements with relatively low deleterious potential and rarely changing elements with high deleterious potential. Random mutagenesis in the latter are not beneficial to the individual, but if kept at sufficiently low rates, it may still be beneficial at the population level.

### Exemplary implementations of adaptive improvisation

Below we propose specific examples of potential mechanistic implementations in both unicellular and multicellular organisms. While these mechanisms may have a significant contribution to adaptation, they are not the only mechanisms which can support adaptive improvisation. We therefore include these mechanisms only as a way of demonstrating the feasibility and generality of the hypothesis and to provide clear (but non-exclusive) examples of variations which satisfy the underlying assumptions of adaptive improvisation.

#### 1. Unicellular examples –

##### 1a. *Adaptive improvisation using mobile genetic elements*

Examination of the rapid world-wide increase in antibiotic tolerance of pathogenic bacteria (commonly thought to be associated with acquisition of resistance conferring genes [87, 88, 92, 93]) reveals all the hallmarks of the proposed principle. Bacterial genomes are subdivided into a “core” genome arranged in chromosomes, and an “accessory” genome consisting of mobile genetic elements (MGEs) such as bacteriophages, pathogenicity islands, chromosomal cassettes, plasmids and transposons [94, 95]. These MGEs can be viewed as “weakly-constrained features” capable of contributing to *de novo* adaptation, which can be inherited by vertical and horizontal gene transfer (HGT) mechanisms [59, 92, 96–98]. Each MGE can also have hundreds of non-identical copies and can account for 25 % of the total DNA content [99]. Unlike the core genome which contains all the constitutively vital genes (metabolism, DNA synthesis etc.), the accessory genome is enriched with genes promoting adaptations to various ecological niches (resistance, virulence factors etc.) [100, 101]. Genetic analysis at a population level shows that MGEs are more variable compared to most regions of the genome [102, 103]. This variability is thought to reflect mutagenic potential [104], high recombination rates [105] and other forms of genetic instability and intercellular exchange of genetic elements [106–108]. Moreover, changes in MGEs or their complete loss, is typically less detrimental to the bacteria compared with chromosomal loss. Accordingly, several studies suggest that HGT of MGEs is a major factor in regulatory evolution [86] and a key contributor to acquisition of bacterial tolerance to toxic stress [92, 97, 109, 110]. Notably, some of these transfer mechanisms are increased or enabled by stress [111], and various classes of MGEs also exhibit stress-dependent increase in the recombination and mutation rates [104, 105].

The acquisition of bacterial stress tolerance by random changes in MGEs and their exchanges between individuals, is also compatible with stabilization of tolerance by positive (auto-regulatory) feedback mechanisms. For example, nutrients and signaling molecules that are induced (directly or indirectly) by beneficial changes in bacterial MGEs, may promote their own production in the altered bacteria as well as in their non-altered neighbors.

In this particular example, adaptive improvisation relies on two distinct carriers of genetic information: A relatively stable, slowly evolving genome which encodes many essential functions, and a flexible genome which is more sensitive to stress, varies on faster timescales, exchanges more readily between bacteria and has reduced potential to harm the bacteria (compared with equivalent changes in the core genome). Under new stressful conditions, bacterial MGEs can undergo many rapid changes, which may alleviate the stress and reduce the drive for further changes.

##### 1b. *Adaptive improvisation via exosome-mediated exchange of biotic materials*

Adaptation by improvisation does not necessarily rely on genetic changes. One example based on diverse types of potential tolerance-conferring factors is provided by production and exchange of exosomes. Many cells release small (30-250 nm sized) extracellular vesicles which can be loaded with diverse set of biomolecules, including DNA, RNA (both coding and non-coding), proteins and other cytosolic components [112, 113]. These vesicles can deliver factors which support growth [114] and enable inter-cellular communication [115, 116], which could allow coordination of cellular activities. Exosome release can be enhanced by stress [117], increase stress tolerance [118–120], assist in promoting or evading immune responses [121, 122], facilitate tumor survival and progression [123–125] and may also protect cells from pathogens [114].

Production and exchange of exosomes between cells increases the overall amount and diversity of changes. Moreover, when a subset of cells produces exosomes with factors that contribute to stress tolerance, the exchange of these exosomes increases the tolerance of recipient cells. If the underlying cargo is also self-enforcing, the increase in tolerance could be substantially enhanced and stabilized. Participation of a large population in exosome production and exchange further allows each cell to acquire tolerance with a lower level of exploration on its part. This benefit of cross tolerance reduces the potential for negative outcomes. As in the case of MGEs, the potential for horizontal transfer of DNA via intercellular exchange of exosomes provides a straight-forward mechanism for transgenerational transfer of the acquired stress-tolerance [126]. The discovery of functional contributions of exosomes in marine ecosystems [114] suggests that they may account for part of the rapid adaptation of corals to climatic change [127].

#### 2. **Multicellular example –**

##### *Adaptive improvisation via random changes in symbionts*

**Fig. 3:**
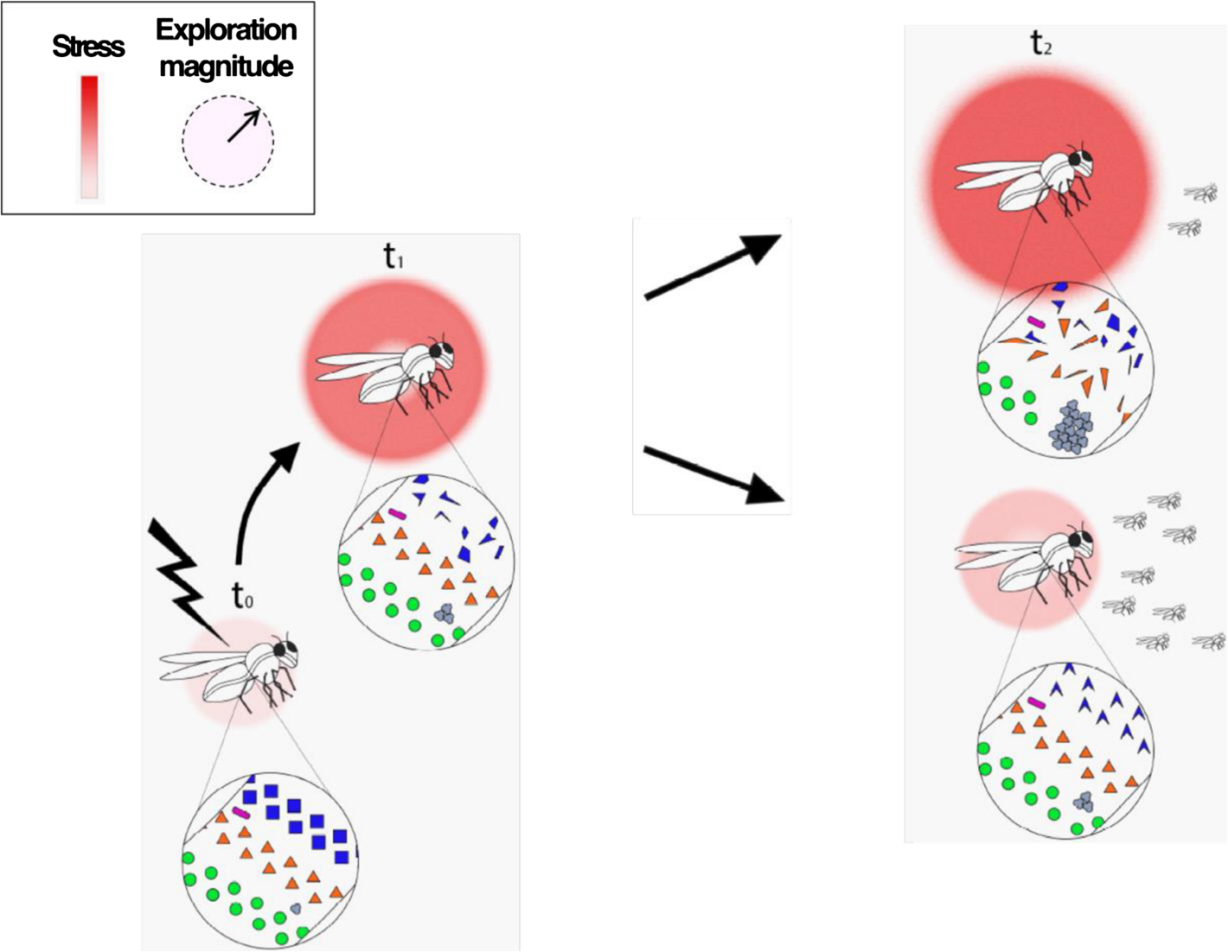
Potential realization of adaptive improvisation by host-microbiome interactions in animals (illustrated here using flies as an example). A novel stress induces rapid changes in the composition of bacterial species, as well in the intrinsic properties of individual bacteria, their spatial distributions and their interactions. The characteristic rate and magnitude of changes (represented by the diameter of the color-coded halos) tend to increase with the strength of the stress (displayed in red color code). Many of these changes occur during the lifetime ‘T’ of an individual (i.e. t_2_ - t_0_ < ‘T’). The modified microbiome influences the state and properties of the host, potentially increasing or alleviating the stress in the host and the holobiont. Changes which alleviate the stress also reduce the drive for further changes, thereby decreasing the characteristic magnitude of subsequent changes (smaller halo). Bacterial species are represented by specific colors. Variation within a particular species (e.g. physiological, epigenetic and certain genetic changes) is indicated by shape modifications without a change in color

A particularly notable (but not exclusive) example in multicellular organisms is based on the symbiotic microbiome. The latter typically includes a very large number of micro-organisms, capable of responding more readily and rapidly to external or internal perturbations (compared with host cells). The contribution of symbionts to the development and homeostasis of their hosts has long been recognized [52, 128–130], and more recently referred to as the holobiont theory [131–133]. While this theory also recognized the potential contribution of heritable symbionts to rapid evolution of their host, it did not address the possibility (or requirement) for frequent emergence of *de novo* adaptations in each individual. Accordingly, it does not specify an organizing principle by which random changes in symbionts can account for *de novo* emergence of individual–specific adaptations during the lifetime of evey individual. We propose that the broad host-microbiome interactions enables stress reduction in the host by random changes in the microbiome. Under stressful conditions which are not efficiently alleviated by pre-evolved responses, the microbiome rapidly undergoes a series of diverse changes (e.g. changes in species composition, gene sequence, epigenetic state, etc.). These changes could be induced by direct exposure of the microbiome to new external environments or indirectly by an input from the stressed host [134]. In a very short period of time (relative to the generation time of the organism), a very large number of micro-organisms can change and produce (or eliminate) many products which could affect the host. If the alterations in the microbiome happen to alleviate the stress, they also reduce the drive for further changes and the new microbiome state is more likely to persist/stabilize (Fig. 3). In this way, the microbiome can act as an adaptive buffer, providing the organism with at least partial solution that reduces negative outcomes for the host and diminishes the drive for varying more stable host-intrinsic traits. It is important to note, however, that induced changes in the microbiome are not guaranteed to reduce the stress. In many specific scenarios, the stress will either remain at similar levels or even increase. However, in these cases, the microbiome will likely continue to change, thereby exploring new avenues for mounting a beneficial response. This should create a statistical bias towards better outcomes compared to those achieved without the feedback, resulting in net probabilistic gain of adaptation within the lifetime of a single individual.

As in the cases of MGEs and exosomes in single cells, beneficial changes in the microbiome of animals can be amplified and stabilized by feedback mechanisms. Random changes in bacteria which happen to reduce the stress and also promote expansion of the altered bacteria (on the expense of other micro-organisms), could lead to further expansion of the beneficial bacteria and reciprocal displacement of less beneficial micro-organisms. The beneficial outcome can also spread in a population of hosts via the transfer of these bacteria.

Regardless of the eventual outcome on the stress, changes in the microbiome affect host-intrinsic processes [128, 131–134]. As such, the changes in the microbiome can promote physiological and epigenetic modifications in the host, which in turn feedback on the microbiome until the stress falls below a threshold for a change (or, alternatively, until the organism dies). Hence, a bacterial-mediated (adaptive) process of stress reduction is expected to induce a gradual change in the host, particularly in its more weakly constrained internal processes and components. We have recently hypothesized that the microbiome can assist the organism in being both robust and plastic by balancing stability and flexibility based on contextual demand [134]. The current proposal extends this idea by suggesting a mechanism in which the robustness of important traits is achieved by the plasticity of less stable ingredients, such as the symbiotic micro-organisms. This highlights the dichotomous contribution of plasticity to both generation and suppression of variation.

The potential heritability of adaptability-conferring changes in the microbiome, provides rich infrastructure for rapid emergence of new adaptive responses. This buildup of adaptive responses is greatly assisted by the accumulation of beneficial changes in every generation of the organism, thus improving the overall response within timescales that are much faster compared to genetic adaptation of the host [132, 134]. Potential examples include the rapid spread of the defensive endosymbiont *Spiroplasma* in *Drosophila hydei* exposed to high parasitoid wasp pressure [135], the regulation of thermo-tolerance in the Aphid *A. pisum* by mutations in its obligatory endosymbiont, *B. aphidicola* [136], the regulation of plant specialization in this Aphid by its facultative proteobacterium [137] and the rapid evolution of competitive, yet suboptimal strains of symbiotic mesorhizobia on the legume *B. pelecinus* [138].

While the microbiome provides a relatively simple and powerful realization for acquiring new adaptations in metazoans, the proposed principle of stress-regulated improvisation in multicellular organisms is not limited to changes in the microbiome. It is broadly applicable to other host-intrinsic factors and processes. Similarly, the MGE- and exosome-based implementations in single cells should not be considered unique and likely occur in parallel to a variety of other mechanisms (e.g. prion-based acquisition of beneficial phenotypes under stress [59]).

### Predictions of the hypothesis

Below we provide a set of predictions derived from various aspects of the hypothesis. It should be realized, however, that some predictions are potentially more informative, especially those which are designed to test if highly similar conditions of novel stress (e.g. in replicated experiments) can result in substantially different adaptive outcomes. Additionally, the dependency of adaptive improvisation on random processes, limits the ability to predict the outcome of every experiment. Accordingly, we use the terms’ expectations’ and ‘tendencies’ in a statistical sense to indicate that a particular prediction should be confirmed by averaging over a sufficiently large set of conditions and experiments, but is not necessarily expected in every setup or every experiment.

#### The extent of adaptive improvisation increases with the strength of stress

All living organisms have a capacity to reduce stress by improvisation within the lifetime of an individual. This capacity co-exists and can be influenced by pre-evolved responses. The rate and extent of the improvisation part of the response is expected to increase with the strength of the stress, which in turn, depends on the state of the organism, its current response and the external conditions. Compromising stress-relieving activities of pre-evolved responses (e.g. inhibiting heat shock response under exposure to high temperatures) will elevate the stress and increase the extent of random changes.

#### The capacity to adapt by improvisation increases with the volume and rate of change of flexible features

For example, organisms with a more diverse gut microbiome are expected to cope better with novel stress compared with the same organism with a limited diversity. Conversely, experimental removal of the microbiome (or reduction in other major sources of flexible variation) is expected to reduce the capacity to withstand novel conditions of stress. These conditions can be established by experimentally perturbing the endogenous regulation in ways which create highly unexpected maladaptive conditions (for example, by ectopic expression of a toxic gene from the promoter of an essential gene, or alternatively, placing an essential gene under the regulation of a silenced promoter [82, 139]).

#### Different processes which can mediate adaptation by improvisation can only be specified in terms of probabilities

The improvisation part of the response is expected to differ substantially between independent biological replicates of the same condition (in contrast to the reproducibility expected from pre-evolved responses). The probability of each outcome depends on the relative fraction of scenarios which lead to this particular outcome.

Initial support of this prediction has been provided by gene recruitment in yeast, showing that emerging adaptations ban be associated with global transcriptional changes which exhibit very little overlap between independent experiments [83, 84, 140].

#### Adaptive improvisation induces new correlations and anti-correlations between and within interacting individuals

For example, induction of a secreted auto-regulatory factor which promotes its own production in both the original producer and its neighbors will stabilize and synchronize the production across the interacting individuals. This coordination will tend to reduce differences between responses of co-exposed individuals compared with individuals in non-interacting populations that are subjected to similar conditions of stress. Similarly, adaptive responses which involve interactions between opposing changes can promote coordinated segregation into two anti-correlated subpopulations. Since the exact same rationale applies to interacting changes within a single individual, adaptive improvisation is also expected to promote correlations and anti-correlations between newly-forming changes within each individual (including within individual cells of a multicellular organism).

This prediction is also consistent with gene recruitment studies in yeast which revealed increase in gene expression correlations and anti-correlations under stress [83].

#### Co-improvisation of interacting individuals decreases the extent of improvisation per individual

Beneficial outcomes of improvisation in a single individual (organism or cell) can be shared with its interacting partners, thereby acting to reduce the average stress per individual. This network contribution to stress-reduction decreases the drive for improvisation per individual.

#### Adaptive improvisation provides a wide range of Lamarckian adaptations

Combining a wide range of adaptive changes that emerge by improvisation with  mechanisms of non-Mendelian inheritance [141], creates a multitude of opportunities to transfer beneficial responses across generations. If newly forming adaptive changes are at least partly heritable, the stress is expected to decrease over successive generations of exposure to an ongoing stressful condition. For example, some of the changes in the gut microbiome which happened to have a positive impact on the host will be inherited by the host offspring. If the stressful conditions persist, the adaptation of the host will tend to improve by additional changes in the inherited microbiome. This processes will accompanied by a progressively reduced likelihood of complete reversal of the microbiome state and will eventually prevent reversal of corresponding traits in the host. While this example is based on changes in the microbiome, progressive improvement by coupling beneficial improvisation with non-Mendelian inheritance is widely applicable to host-intrinsic factors and does not depend exclusively on microbiome changes.

#### Adaptation by improvisation is slower than adaptation by pre-evolved responses

Because of the exploratory nature of adaptive improvisation, alleviation of stress by exploration will typically require a longer period of time compared to responses which have been previously selected based on their ability to accommodate this stress (e.g. pre-evolved responses to recurrent stress such as heat shock). A novel scenario of stress is also expected to delay ongoing processes. For example, a novel stress during a particular stage of animal development will tend to prolong this stage.

#### Stress reduction by adaptive improvisation occurs simultaneously in multiple levels of organization

As a basic principle of biological self-organization, we expect the occurrence of adaptive improvisation in every stage and level of organization of living systems. These include the development, function and behavior of unicellular and multicellular organisms at any scale and arrangement (e.g. free living micro-organisms, symbionts, animal and plant cells, tissues and organs, complex societies of unicellular and multi-cellular organisms and larger ecological systems). Adaptive improvisation at any level can induce changes in any other level. For example, stress that is initially triggered in a specific cell within a tissue, will not necessarily remain restricted to this cell; a sufficiently strong stress will drive intracellular changes which affect nearby cells, thus leading to a tissue-wide stress. Likewise, a strong enough tissue-wide stress would scale up to the organ level. Similar scale-ups will lead to systemic, organism-wide changes which could further affect the external environment and the neighboring organisms. At every scale, the extent of adaptive improvisation will correspond to the degree and persistence of stress in this level. These interactions will tend to coordinate co-occurring variations in different locations and scales, leading to simultaneous stress reductions at multiple levels.

#### Adaptive improvisation per cell is lower in multi-cellular vs. unicellular organisms and higher in dysregulated vs. normal cells

These predictions stem from the expected repressive effect of constraints on the ability to improvise. To maintain functional organization of a multi-cellular organism, the spectrum of deviations per cell needs to be narrower than in unicellular organisms. Without a sufficiently strong restriction (constraints) on the spectrum of deviations, adaptive improvisation in individual cells is more disruptive to coordination between cells, tissues and organs. This is much less of a problem in unicellular organisms, because the survival of each individual is less dependent on its degree of coordination with other cells. Unicellular organism are therefore more ‘free’ to adapt by improvisation compared to cells within animals.

#### Reduction of cellular plasticity compromises the capacity of tumors to resist treatment

Much of the constraints which restrict cellular changes that are harmful to a multicellular organism, are embedded in the pre-selected gene regulation of this organism. These constraints are compromised under conditions of dysregulation such as in tumorigenesis. Removal of constraints in these cells increases their capacity to adapt by improvisation and reduces their coordination with other cells in the tissue. This makes these cells less cooperative with the functional integrity of the whole organism. In that respect, tumor cells behave like unicellular organisms [58], which undergo rapid and relatively uncoordinated adaptations to new environments within the whole organism, eventually leading to its destruction. Since adaptive improvisation can be achieved by a wide variety of mechanisms, it provides tumor cells with an unusually broad adaptive potential which is not exclusively dependent on a single mechanism. This can account for the ability of tumors to resist a vast array of cancer treatments. We therefore expect a much larger efficacy of treatments which combine anti-tumor drugs with factors that limit the capacity of cells to adapt by improvisation (general factors which reduce cellular plasticity).

#### Adaptive improvisation can provide man-made systems with ability to overcome ‘new problems’

Here adaptive improvisation refers to reduction of stress-like observables analogous to the above notion of biological stress. New problems, in turn, correspond to internal or external failures which compromise the durability of the system and for which a troubleshooting solution has not been built into the original design. For example, implementation in self-reproducing machines [142] may enable generation of much more complex automata with restorative, replicative, and evolvable capacities. Similarly, implementation in artificial intelligence systems might extend their capacity to solve problems that were not initially taken into consideration.

### Implications of the hypothesis

#### Adaptive improvisation as a complement for natural selection at the single individual level

This principle of self-organization offers a conceptual process enabling adaptation to novel stressful conditions occurring during the lifetime of every single individual. As such, it addresses a critical limitation of natural selection and specifies an efficient new way in which physiology might contribute to evolutionary processes [50, 143, 144]. The combination of restricted variation in potentially deleterious directions and feedback between variation and stress, explains how any type of newly-forming random variation can self-organize into emergent physiological adaptations. Since some of the adaptive changes are also heritable, this proposal principle provides a robust path towards highly broad Lamarckian-like adaptations which are not exclusively dependent on a specific choice of pre-evolved mechanism. The connection between emergent physiological adaptations and progressive improvements within few generations further imply that evolution should be regarded as occurring on every temporal scale and is not limited to changes in allele frequencies.

#### Stress as an organizer of random variation into adaptive outcomes

The proposed picture implicates stress not only in driving newly-forming variation, but also in constantly organizing variation into adaptive outcomes. The above notion of stress is, however, substantially different from the view of stress as activation of a defined stress pathway. Here, we regard stress as a collection of driving forces which operate on diverse processes across multiple scales (from the scale of intracellular processes, through tissues and organs, and up to the whole organism level and above). Regarding stress in this way: 1) accounts for a tendency to re-achieve broad stability (drive reduction), and 2) explains how *de novo* coordination between emergent adaptations in cells, tissues and organs can arise by parallel stress-reductions in multiple scales.

#### Modified picture of gene-regulation and evolution

This proposal portrays gene regulation, development and evolution as a synergy between outcomes of natural selection and ongoing adaptation by improvisation. The latter may be essential for viability, durability and evolvability, and it is possible that no organism can exist without it. The extent of adaptive improvisation increases with the magnitude of stress that was not alleviated by pre-evolved mechanisms. This provides a natural ‘division-of-workload’ between pre-evolved process and adaptive improvisation, without a need for a computational-like ‘decision making’ process. Similarly to natural selection, adaptation by improvisation can involve any kind of mechanism. The principle of adaptive improvisation therefore extends adaptation by natural selection to temporal and organizational scales within single individuals.

#### Origin of tumorigenesis

Adaptive improvisation can explain why dysregulation leads to formation of heterogeneous tumors which detach from normal coordination and become extremely resistant to drugs. It suggests a modified strategy of cancer therapy which would compromise the improvisation capacity of tumor cells by making use of factors which reduce cellular plasticity.

#### Generalization

As a fundamental principle of organization, adaptive improvisation by drive-reduction may be implemented in complex systems beyond life sciences (e.g. in physics, engineering and economy), thus improving the ability of these systems to overcome arbitrary types of novel malfunctions.

## Reviewers’ comments

### Reviewer’s report 1: Prof. Eugene Koonin

The article by Soen, Knafo and Elgart discusses “adaptive improvisation” which the authors portray as a novel principle of evolution driven by unfamiliar (i.e. not encountered previously) forms of stress. In my view the paper does not quite live up to such dramatic claims. The term of adaptive improvisation is new but the specific examples of such “exploratory response” presented by the authors, namely stress-induced mobilization of MGE, DNA transfer by exosome, and changes in the microbiome, are well known. I do agree that these mechanisms (and other similar ones, e.g. stress-induced mutagenesis) can be legitimately described as exploratory response and do represent a distinct evolutionary modality (in Ref. 59, Yuri Wolf and I defined this class of phenomena as quasi-Lamarckian). If this is all that is meant, the proposition is not novel. If there something more to “adaptive improvisation”, the present text is too vague to discern these potential new aspects. To my regret, the proposed tests of the hypothesis are also presented vaguely, and at least from the current version of the paper, I could not figure out what if any specific experiments or analyses are proposed. As it stands, the article is, in my opinion, not a hypothesis but an essay but even in that capacity, is unnecessarily opaque and not sufficiently specific.

I cannot rule out that I missed the more novel and concrete aspects of the hypothesis. If that is the case, however, I think the authors should make an effort to present their ideas simply and clearly.

Quality of written English: Needs some language corrections before being published.

Authors’ response: *We revised the manuscript (and its title) to provide a clearer description of the hypothesis. Below we emphasize the novel elements of this hypothesis. To avoid potential confusion, it is important to keep in mind that stress-mediated induction of variation (heritable or not) is not the subject of this hypothesis. The provided mechanistic examples (*e.g. *MGEs, exosomes and symbiotic microorganisms) are also not the subject. We include these mechanisms only as a way of demonstrating the feasibility and generality of the hypothesis and to provide clear (though non-exclusive) examples for the kind of variations which satisfy its underlying assumptions. The hypothesis is first and foremost about a fundamental principle of organization and its ability to address an adaptation problem that has not been previously discussed, namely: How****every individual****can acquire****diverse physiological****tolerance to****high load****of novel combinations of****internal****perturbations that are****individual****-****specific****(distinguishing features of this problem are highlighted in boldface).*

*While addressing this new problem, the hypothesis indeed use key elements that were previously suggested to contribute to Lamarckian evolution (Koonin & Wolf,* [59]*). It is therefore important to explain the distinguishing features of the current hypothesis. The aforementioned previous publication discussed a framework of co-existing Lamarckian-like and Darwinian mechanisms. It suggested that Lamarckian-like acquisition of heritable adaptation to novel environments can be achieved by stress-induced mutagenesis. It also proposed that the deleterious potential of random mutagenesis is addressed by regulation which involves DNA repair and particular structure of genome architecture (in prokaryotes). The latter are thought to bias mutations in a way which allows coordinated evolvability of functionally linked genes in rare cells where beneficial mutations emerge. Although we agree with this view, it does not address the conceptual problem which we propose to solve, namely: how any regulation could use mostly non-beneficial variation to provide every individual with a beneficial response to arbitrary types of novel conditions. This becomes far more challenging when each multicellular individual has to cope with many (individual-specific) combinations of internal perturbations (or ‘failures’) in different cells, tissues and organs. As we explain in this paper, every individual encounters many of these failures during its lifetime even under regular environments. Thus, the difficulty is not in explaining how variation can be induced or inherited. The problem is to explain how regulation within an individual can organize variation into emergent adaptations to novel scenarios of diverse kinds. A solution which is based on random variation must also explain how deleterious effects of non-beneficial variations are avoided and how emergent adaptations in different cells, tissues and organs are coordinated into a coherent benefit for the whole organism. Without such ability to organize variation in a beneficial way, the benefit of inheriting this variation is also questionable. While genome architecture and DNA repair likely participate in the solution (as are other mechanisms), the conceptual problem remains unanswered. Moreover, substantial part of the variation which can participate in emergent physiological adaptations is not at all genetic. Since our hypothesis focuses on providing a conceptual solution to this yet unaddressed organizational/regulation problem, its core message does not overlap with prior suggestions.*

*The proposed solution (Adaptive Improvisation) is achieved by incorporating a principle of organization which is not part of current evolutionary thinking and is not exclusively dependent on any specific mechanism. It explains how large volume of random variation of any kind (including, but not limited to genetic variation) and every scale (from intracellular to the whole organism) can be safely organized de novo into many beneficial physiological adaptations during development and homeostasis of every single individual. This should not be confused with previously reported examples of Lamarckian adaptations by pre-evolved mechanisms which address a specific type of novelty. Hallmark examples are the CRISPR system in prokaryotes* [69–71] *and the small RNA-based mechanism of viral immunity in C. elegans* [72]*. Both mechanisms enable rapid acquisition of heritable resistance to new viruses, but the tolerance is always acquired using the same mechanistic pathway. While the preexistence of such pathway is very instrumental for coping with new viruses, it does not provide solutions for all other types of novel conditions (including those which can arise as outcomes of new pathogenic viruses that have managed to evade rapid elimination). This highlights the need for an adaptive approach which can use arbitrary mechanisms in conjunction with any kind of new variation at any scale.*

*Another major and critical distinction with respect to prior discussions is the perception of stress. Specifically, the stress in this hypothesis is not necessarily regarded as the activation of a defined stress pathway. Rather, it is viewed as deviations from steady states (or disruption of ongoing processes) which are subsequently capable of driving more deviations (with or without activation of specific stress pathways). In this new view, stress is a collection of driving forces which operate on diverse processes across multiple scales (from the scale of intracellular processes, through tissues and organs, and up to the whole organism level and above). Regarding stress as a mere driver of variation (analogously to physical stress) is required to: 1) account for a tendency to re-achieve stable states and processes of any kind (drive reduction), and 2) enable coordination between adaptive changes which take place across different scales of a multi-cellular organism. It is important to note that de novo emergence of adaptations in cells, tissues and organs are not enough for coping with diverse internal failures. Such coping also requires that the coordination between adaptive outcomes in different scales will also emerge de novo. This cannot be achieved by pre-specified mechanisms of coordination. Yet, by regarding stress as a driver of variation in every scale, coordination can arise as an outcome of stress-reductions which occur in parallel over multiple scales.*

*The necessity of ‘real time-like’adaptive capabilities beyond natural selection leads us to propose a new picture of gene regulation as (an inseparable) synergy between outcomes of natural selection and adaptation by improvisation. To prevent confusion, we do not just mean to suggest that adaptive improvisation is merely assisting natural selection. Rather, we argue that the dependence of every individual on its ability to address high load of novel internal failures, means that natural selection and adaptive improvisation are inter-dependent. Simply put, one is insufficient without the other. Similarly to natural selection, adaptation by improvisation can involve any kind of mechanism. To further establish this view, the hypothesis explains how the workload can be divided between pre-evolved responses and adaptive improvisation without any need for a ‘decision-making’ process. Here again, stress is the key factor; the less it is reduced by outcomes of natural selection, the more it drives improvisation-based processes. In this new picture, stress is a major physiological organizer of variation, whereas fitness is the combined outcome of natural selection and adaptive improvisation. Note that the stress in this picture cannot be reduced to zero, because it is constantly influenced by two opposing factors: it is increased by the 'entropic' effect of the higher abundance of non-beneficial (*vs. *beneficial) variations and it is reduced by (less frequent) beneficial variations.*

*As a fundamental principle, this hypothesis has broad implications which can be translated into many predictions (and applications). However, unlike hypotheses which are based on one or a few deterministic mechanisms, our proposal is inherently based on largely random organizations which involve a wide range of processes and mechanisms. This limits the ability of verifying or falsifying the hypothesis using a single experiment. Of course, some evaluations are potentially more informative, especially those which are designed to test if coping with similar conditions of novel stress (*e.g. *in replicated experiments) can result in substantially different adaptive outcomes. Notwithstanding, we believe that sufficient level of confidence would ultimately require different types of analyses, each focusing on one or few predictions of the kind provided in thismanuscript.*

*For completeness, the paper also discusses the potential relevance of adaptive improvisation beyond life-sciences (*e.g. *in man-made systems).*

## Reviewers’ comment

I appreciate the extended response given by the authors to my comments. I must admit that the level of abstraction in this discussion remains largely above my head. Hopefully, other, theoretically better versed readers receive inspiration from this treatise.

Quality of written English: Acceptable.

### Reviewers’ report 2: Dr. Yuri Wolf

Soen, Knafo and Elgart touch upon an important topic of the nature of adaptation to novel challenges. To me, however, this discussion seems to be a bit too vague and general.

Quality of written English: Acceptable

Authors’ response: *As indicated above, we revised the manuscript (and its title) to provide a clearer description.*

## Reviewers’ comment

The authors improved the presentation, but the main problem remains - in my opinion it is impossible to criticize or embrace ideas, formulated in such a general terms. Even the very existence of a problem in need of solution is not entirely obvious - yes, none of the generic plasticity mechanisms can fully eliminate all the problems (p.4), but at the same time not all and every individual of the population must survive for the population to survive. Without a careful quantitative analysis it is difficult to understand which of the proposed novel entities (principle of Adaptive Improvisation, self-stabilization of stress-reduction, etc.) are driven by necessity.

Quality of written English: Acceptable.

### Reviewers’ report 3: Dr. Itai Yanai

This reviewer provided no comments for publication.

## Abbreviations

MGEs: Mobile genetic elements
